# The optimization of the diagnostic work-up in patients with suspected obstructive lung disease

**DOI:** 10.1186/1471-2466-10-60

**Published:** 2010-11-23

**Authors:** Frank J Visser, Milena JMM van der Vegt, Gert Jan van der Wilt, Julius P Janssen

**Affiliations:** 1 Canisius Wilhelmina Hospital, Nijmegen, The Netherlands Department of Pulmonary Medicine, Canisius-Wilhelmina Hospital, Nijmegen, The Netherlands; 2 Department of Epidemiology, Biostatistics, and Health Technology Assessment, Radboud University Hospital, Nijmegen, the Netherlands

## Abstract

**Background:**

Pulmonary function testing is a key procedure in the work-up of patients who are suspected of having asthma and chronic obstructive lung disease (COPD). Therein, clinical visits and pulmonary function tests (PFTs) are the major contributors to the overall financial costs.

The aim of this study was to assess whether a specific diagnostic test protocol contributes to the optimization of the work-up of patients who are suspected of having asthma and COPD.

**Methods:**

A prospective, single-blind, and randomized controlled study was performed. In the control group (CG), all of the PFTs that were ordered by the lung specialist were carried out. In the experimental group (EG), specific PFTs were selected according to our protocol. The primary end point was the total cost of achieving a final diagnosis.

**Results:**

One hundred and seventy-nine patients were included into this study: 86 in the CG and 93 in the EG. The mean number of tests to diagnosis was 3.8 in the CG versus 2.9 in the EG (P < 0.001). The mean number of redundant PFTs before diagnosis was 1.2 in the CG versus 0.08 in the EG (P < 0.001). The number of patients who required an additional outpatient visit to complete diagnosis was higher in the CG in comparison to the EG (P = 0.02). The mean cost of work-up per diagnosis was €227 in the CG versus €181 in the EG (P < 0.001).

**Conclusions:**

In this group of patients with suspected obstructive lung disease, protocol-driven, PFT-based selection is more cost-effective than test selection at the discretion of lung physicians.

## Background

Diagnosing asthma and COPD is an important part of the daily practice of pulmonary physicians. Pulmonary function tests (PFTs) play a key role in the work-up of obstructive pulmonary diseases [1-3].

No exact figures exist for the annual costs that are associated with current diagnostic processes, although they are likely to be substantial.

**Figure 1:**
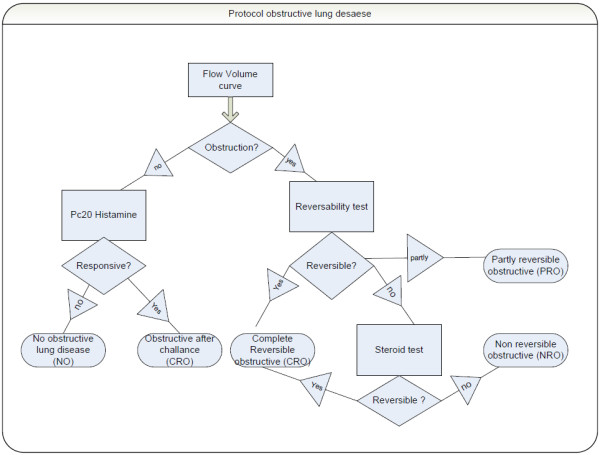
**Pulmonary function protocol for obstructive diseases**. Criteria for obstruction, airway responsiveness (PC20 histamine), reversibility, and steroid tests (see text).

Finding the optimal diagnostic work-up in patients with obstructive lung disease is challenging. A physician who routinely orders most or all PFTs in the work-up of asthma and COPD patients runs the risk of unnecessary testing; however, a physician who orders tests more sparingly runs the risk of unnecessary outpatient visits. In view of the high incidence of patients with obstructive lung diseases, it is important to find the optimal diagnostic work-up in each of these patients. To this end, we have developed a diagnostic protocol (Figure 1) that can be jointly used by physicians and pulmonary function assistants. In our group, some physicians already use this diagnostic PFT protocol; however, some of the physicians order PFTs without following the prescribed diagnostic PFT protocol. Prior to the beginning of this study, there was no available evidence that demonstrated that protocol-driven PFT ordering is more efficient than physician-driven test ordering. Therein, we hypothesize that protocol-driven test ordering will be more efficient than test ordering without direction from a diagnostic protocol.

The aim of this study was to assess whether protocol-driven test ordering reduces the number of redundant pulmonary function tests, decreases the number of outpatient visits, and increases the cost effectiveness of patient work-up in comparison to physician-driven test ordering.

## Methods

A prospective, randomized, and single-blind trial was conducted at our outpatient unit.

An institutional review board (IRB) approved this study. This study was only a formal stratification of the current practice; hence, informed consent was not necessary.

## Methods

The study participants consisted of consecutive adult patients who were referred to our respiratory outpatient clinic and suspected to have asthma or COPD at the end of the first outpatient visit. None of the patients had recently (in the preceding three years) been diagnosed with asthma or COPD by a pulmonary physician. Patients were primarily referred by general practitioners; however, two patients referred themselves to our outdoor department, and three were referred by cardiologists. We excluded patients who were not able to adequately complete pulmonary function tests, were referred to a pulmonary physician because of an abnormal X-ray, needed pre-operative consultations, and have had an infection of the upper or lower airways or a possible exacerbation of obstructive airway disease in the past two months.

In the first visit, the physician takes a medical history, performs a physical examination, makes a differential diagnosis, and orders laboratory testing, such as a chest X-ray. Only patients who were most likely to be diagnosed with asthma or COPD were included into this study. Physicians ordered diagnostic tests as they deemed appropriate and added the reason for the pulmonary function testing (e.g. suspected obstructive lung disease). At the end of the first outpatient visit, nurses randomized the patients into the control group (work-up at the discretion of the physician) or the experimental group (work-up in accordance with the protocol) by pulling an opaque envelope.

PFT lab assistants were notified of the outcome of the randomization in order to allow them to perform investigations as ordered or per protocol.

The PFT- protocol is shown in Figure 1. Therein, when the forced vital capacity (FVC) is less than normal, a total lung capacity (TLC) measurement was conducted in order to exclude restrictive lung disease. When the patients smoked more than 10 pack years, then their respective diffusion capacities were measured.

A second physician independently examined the results for each patient and classified the patients as follows:

### Completely reversible (CR)

(1) An airway obstruction that is completely reversible (FEV1 reversible by ≥ 9% of the predicted value) to a normal range after beta-2 agonist and anticholinergic treatment; patients who received reversibility testing were tested for both bronchodilators; (2) An airway obstruction that is completely reversible after 14 days of 30 mg/day of prednisone; and (3) normal PFTs but a decreased PC20 histamine threshold.

This PFT group supports to the diagnosis of asthma.

### Non-reversible obstructive (NRO)

Reduced FEV1 and FEV1/FVC values, which are irreversible after beta-2 agonist and/or anti-cholinergic treatment (an FEV1 increase of < 9% of the predicted value) and no return to the normal range after 10 days of 30 mg/day of prednisone.

This PFT group supports the diagnosis of COPD.

### Partly Reversible Obstructive (PRO)

Reversibility is present, but an airflow limitation persists. FEV1 increases by ≥ 9% of the predicted normal value but does not return to a normal range after bronchodilators.

This PFT group supports the diagnosis of asthma with persistent airflow limitation or COPD with partly reversibility.

### Normal PFT group

No airflow limitation and a normal PC20 histamine threshold.

This group does not support the diagnosis of obstructive lung disease; hence, a different diagnosis must be considered.

The second physician assessed if the appropriate tests were conducted according to the diagnostic flow and decided on the pulmonary function classification. He assessed the decision about the final diagnosis of the PFT-referring physician.

The second physician calibrated his findings with the findings of the PFT-ordering physician only if there were conflicting findings. In all of these cases, we achieved a consensus on the final diagnosis.

COPD and asthma were finally diagnosed by the first physician on the basis of medical history (smoking behavior, allergies, a family history of asthma, and/or a pre-existing childhood condition); PFTs and clinical investigations, such as the eosinophil count; and the radioallergosorbent (RAST) test.

In the protocol, the criteria for obstruction included an FEV1 < normal and an FEV1/FVC < normal according to Quanjer et al. [4]. Airway hyperresponsiveness was defined as PC20 histamine < 4 mg/ml [5]. Reversibility was defined as a ≥9% improvement in the FEV1 in comparison to a predicted normal value [6-8]. Steroid tests consisted of 30 mg/day of prednisone for 10 days with the intent of reversing the FEV1 to normal levels, as advised by the Dutch Committee: diagnosis for asthma and COPD [7].

Forced vital capacity (FVC), forced expiratory volume in one second (FEV1), and airway responsiveness (PC20 Histamine) were measured according to ERS criteria [1].

During the follow-up visit, the results of all of the investigations that were carried out at the discretion of the physician or according to the protocol were available to the physician who then decided whether a final diagnosis could be made. Follow-up visits and additional PFTs were scheduled as deemed appropriate.

Redundant PFTs were defined as tests that were not absolutely necessary to establish a final diagnosis. For example, a reversible obstructive PFT made the histamine provocation test redundant, whereas a normal flow volume curve made the reversibility test redundant.

The economic analysis was conducted from a health care perspective that included only direct medical costs. Where available, unit cost prices were derived from a national guideline for the economic analysis of health care services [9]. In other instances, real cost prices were calculated on the basis of hospital administration data (Table 1).

**Table 1:** The cost of PFTs and follow-up visits

Tests	Cost
Flow volume curve	€15,00
Reversibility (bronchus dilators) testing	€18,00
TLCO (diffusion capacity)	€41,00
Hyper reactivity (histamine)	€84,00
TLC	€41,00
Outpatient visit	€41,00
Steroid test	€33,00

### Statistical analysis

The unpaired t test with Welch's correction was used to test for the statistical significance of differences between the two groups. An alpha of 0.05 or less was considered to be significant. For statistical calculations, we used GraphPad Prism5 for Microsoft Windows http://www.graphpad.com.

## Results

From a total of 183 patients, 179 patients were included in this study: 86 patients in the CG and 93 patients in the EG. Four patients were excluded for the following reasons: one patient had a malignancy, one patient failed to follow-up, one patient died within a week of the start of the study, and the protocol was not followed with one patient. The second physician calibrated his findings with the findings of the PFT-ordering physician only if there were conflicting findings. In all of these cases, we achieved a consensus on the final diagnosis.

Table 2 summarizes patient characteristics at baseline. Classification of PFT groups on the basis of PFT results and final diagnoses are presented in Table 3. In the control group, 35 patients were diagnosed with asthma versus 39 patients in the experimental group. For the non-reversible obstructive group, these numbers were eight and nine patients, respectively. For the partly-reversible obstructive group, these numbers were 12 and 14, respectively.

**Table 2:** Patient characteristics

	Control group	Experimental group
Number of patients	86	93
Avg. age (years)	55.4	56.8
Sex (% male)	45	46
Height	168.4	170.8
FEV1% pred.	77.8	78.0
FVC % pred.	91.3	91.0
Non-smoker	41%	38%
Ex-smoker	20%	23%
Current smoker	33%	29%
Unknown smoker	6%	10%

**Table 3:** Classification on the basis of PFT results and final diagnoses

Classification on the basis of PFT	Control group	Experimental group
Completely reversible obstructive (CRO)	41%	42%
Non-reversible obstructive (NRO)	17%	17%
Partly reversible obstructive (PRO).	14%	15%
Normal PFT (NO)	27%	26%
Final diagnosis		
Asthma	41%	42%
Asthma partly- reversible	5%	1%
COPD non- reversible-	17%	17%
COPD partly- reversible	10%	14%
Other diagnosis	27%	26%

Diagnosis in the normal PFT group consisted of sarcoidosis (1), gastroesophageal reflux (GER) (5), rhinitis and/or sinusitis (7), hyperventilation syndrome (3), persistent cough (15/21 were smokers or ex-smokers), and dyspnea (3/8 were smokers).

On the basis of clinical assessment, 52 patients were found to have COPD with the following classifications: 7 patients in GOLD stage 1, 24 patients in stage 2, 13 patients in stage 3, and 8 patients in stage 4.

The total cost of the procedures that were used to reach a final diagnosis in patients who were suspected of having obstructive lung diseases.

**Figure 2:**
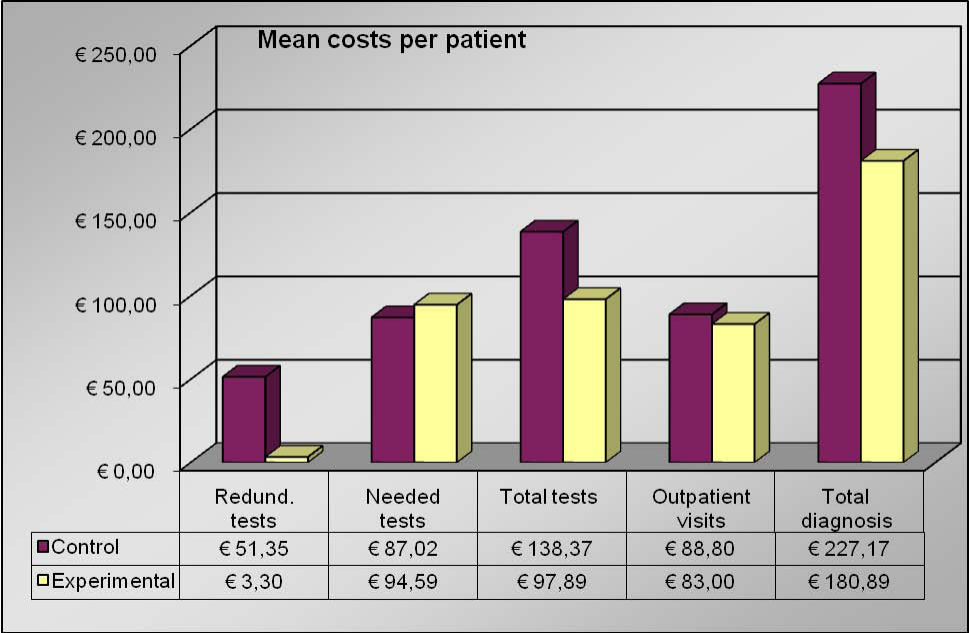
**Mean cost per patient per diagnosis**. Redund. = Redundant.

In the control group, the mean total cost of testing and outpatient visits per diagnosis were €227.17, whereas, in the experimental group, this cost was €180.89 (Figure 2, P < 0.001), which is a 20% reduction in cost.

### The number and cost of outpatient visits until diagnosis

In the control group, two outpatient visits were needed to reach a final diagnosis in 71 patients, whereas 15 patients needed three outpatient visits (mean 2.17, median 2). In the experimental group, 90 patients had two visits, and 3 patients had three visits (mean 2.03, median 2). The difference in total visits between these groups was statistically significant (P = 0.02). Mean costs were €88.80 in the control group and €83.00 in the experimental group (Figure 2, P = 0.02).

The number and cost of the PFTs that were needed for diagnosis:

In the control group, a mean number of 3.81 PFTs per patient was necessary in order to diagnose asthma or COPD. In the experimental group, a mean number of 2.94 tests was necessary (Figure 2, P < 0.001). In the control and experimental groups, the mean costs of PFTs were €138.37 and €97.89, respectively (P < 0.001).

### The number and cost of the redundant PFTs that were used for diagnosis

In the control group, a mean of 1.20 redundant PFTs per patient were performed. In the experimental group, a mean of 0.08 unnecessary PFTs were performed (P < 0.001). The mean costs of redundant tests for diagnoses in the control and experimental groups were €51.35 and €3.30, respectively (Figure 2).

Histamine provocation tests and the added cost of reversibility testing were the two most important sources of redundant costs (Table 4).

**Table 4:** Redundant PFTs

Redundant PFT	Control group		Experimental group	
	**Number (%)**	**Cost in €**	**Number (%)**	**Cost in €**

Histamine provocation test	19 (22)	1596	1 (1)	84

Reversibility testing	30 (35)	540	1 (1)	18

TLC	30 (35)	1230	3 (3)	123

Diffusion capacity for CO	24 (28)	984	2 (2)	82

Totals	103 (120)	4323	7 (8)	307

### Time until final diagnosis

In the control group, a mean number of 33.02 days was necessary to reach a final diagnosis, whereas in the experimental group, a mean number of 35.94 days was needed (P = 0.51).

### Post-hoc

We evaluated the added value in making a diagnosis of asthma using the steroid test in our patients. Conforming to the protocol, we needed 11 steroid tests in the control group and 10 steroid tests in the experimental group. Patient diagnoses did not change with the addition of these steroid tests.

## Discussion

The main finding of our study is that the introduction of a problem-oriented protocol for ordering PFTs in patients with suspected obstructive pulmonary disease can reduce the number of redundant PFTs and outpatient visits, which results in a 20% decrease in costs without an increase in time to final diagnosis. Given the high frequency of PFT usage for the diagnosis of obstructive lung disease, this observed decrease in cost results in a substantial savings at a population level. In our practice of 600 patients per year with suspected obstructive lung disease, protocol-guided test ordering can lead to an annual cost reduction of €27,768 = €46.28 per patient. The most important part of these potential savings is a reduction in the need for reversibility testing when a normal flow volume curve is obtained and a reduction in the need for the time-consuming and unpleasant hyperresponsiveness (PC20 histamine) test when obstruction with reversibility is obtained.

Our lung function protocol is based on the asthma and COPD guidelines [7] of the Netherlands and is within the ERS and ATS standards [3,4,6,10]. Therefore, many other countries can use our protocol with slight modifications. Our patients with asthma and COPD are demographically similar to other western European countries, the USA, and Canada. The only difference is that most patients were referred to us from a family doctor, as is typically the case in the UK; however, in some other countries, a family physician may be skipped more often. Therefore, a minor selection bias is possible; however, the diagnostic criteria for COPD or asthma do not depend on the patient's physician. Of course, the skills and the tools are different between general practitioners (GPs) and pulmonary physicians; however, for this study, we included only the most basal lung function tests and omitted tests, such as exercise testing. These tests are not always needed to confirm a diagnosis of asthma or COPD. We believe that the protocol discussed herein could easily be followed by GPs or hospital physician assistants so long as they have access to these basic tests, thus, leading to potentially more health-care savings.

As we stated before, some physicians have used the protocol-driven lung function protocol that we developed approximately three years prior to this study. The author of this study and two other physicians have primarily followed the protocol-driven testing strategy. Two other physicians (one senior and one junior) did not implement this protocol-driven testing strategy because they were not convinced of the ability of this protocol to save time and cost.

We all agreed to perform this study, and the behavior of these two physicians did change after the completion of this study.

Trainees stay for four years in our hospital, and, at the time of the study, we had five trainees in all stages of their educational processes. We asked our trainees to order according to a test-protocol rather than at their own discretion. We advised them that this would be more efficient.

The protocol allows pulmonary function assistants to work more efficiently, which decreases the frequency that they need to interrupt the doctor, who will often be in a consultation. The workflow is based on our national guideline, which resembles the international guidelines of the ERS.

Steroid tests would not have added value to the diagnostic workflow in our patient group; hence, we doubted the need for such a test in a routine setting. After this study, we removed the steroid tests from the PFT protocol.

The total time in days to diagnosis was not different between the two groups; however, without waiting lists for the outpatient department and for PFT's, we believe there will be a difference in time between the CG and the EG that favors the EG.

We want to emphasize that international guidelines and several national guidelines do not recommend reversibility testing as a means to distinguish asthma from COPD, other than when lung function returns to normal limits. We used three pulmonary function groups as an intermediate; however, the final diagnosis of asthma or COPD (or both) can only be made when the full clinical context, in which PFTs are only a part, is considered.

The prediction threshold of 9% is not a commonly accepted threshold for distinguishing asthma from COPD. We only use this criterion to distinguish between the "non-reversible obstructive" and "partly-reversible obstructive" PFT groups. Internationally, this is merely one method that can be used to make this distinction between these subgroups, and no consensus is available regarding which criterion is the best [6].

A weakness in our study is the potential of a Hawthorne effect. The knowledge that they were involved in a trial may have affected the physicians' behavior (test ordering, making a final diagnosis). The only way to avoid this problem is to retrospectively conduct the study, which would challenge its internal validity. Therefore, we decided to conduct a random, parallel design so as to ensure internal validity (equivalence between groups, minimal likelihood of confounding); however, to the extent that our study suffered from a potential Hawthorne effect, this will most likely have resulted in an under-estimation of the impact of protocol-guided test ordering.

In order to minimize the impact of a potential Hawthorne effect, we discussed all of the results within our group after the inclusion period. Some doctors wanted to minimize patient return visits and ordered tests, which were regarded to be unnecessary during the second visit.

## Conclusion

Problem-orientated PFT ordering significantly reduces the number of PFTs, the total cost, and the number of outpatient visits in the diagnosis of asthma and COPD.

## Competing interests

The authors declare that they have no competing interests.

## Authors' contributions

FV: initiated the lung function diagnostic protocol, helped with the data collection, supervised the lung function analyses, and drafted the manuscript except for the first version. MV: corresponded with the Hospital science office about the need for a medical ethical committee, collected the data, and wrote a first draft of the manuscript. GW: advised about the methods and statistics that were used and drafted the study protocol. JJ: initiated the study, participated in the design of the study, and edited subsequent versions of the manuscript. All authors have read and approved the final manuscript.

## Pre-publication history

The pre-publication history for this paper can be accessed here:

http://www.biomedcentral.com/1471-2466/10/60/prepub
